# Paradoxical Anti-diuretic Effects of Thiazide and Thiazide-Like Diuretics in Diabetes Insipidus: A Systematic Review

**DOI:** 10.7759/cureus.82247

**Published:** 2025-04-14

**Authors:** Anne Laure A Perrine, Driti Reechaye, Indrajit Banerjee, Jared Robinson, Indraneel Banerjee

**Affiliations:** 1 Internal Medicine, Sir Seewosagur Ramgoolam Medical College, Belle Rive, MUS; 2 Pharmacology, Sir Seewoosagur Ramgoolam Medical College, Belle Rive, MUS; 3 Surgery, Sir Seewosagur Ramgoolam Medical College, Belle Rive, MUS; 4 Urology, Penn Highlands Healthcare, Pennsylvania, USA

**Keywords:** antidiuretic, diabetes insipidus nephrogenic, diabetic insipidus, mode of action, neurogenic diabetes inspidus, thiazide diuretics, thiazide-like diuretics

## Abstract

Diabetes insipidus (DI) is a rare endocrine disorder predominantly characterized by polyuria, polydipsia, and dehydration, which can be defined as an excess of 3L of urine excretion in an adult over 24 hours, with the urine osmolality being less than 300 mosm/kg H_2_O. Increased water loss from the body can lead to significant complications, including death, from severe dehydration. Hence, timely treatment of the disease to prevent and replace the fluid loss should be instituted.

Thiazide diuretics are the drugs used and act paradoxically in this particular situation. Thiazides and thiazide-like drugs have shown promising results in reducing the volume of urine output and enhancing urine osmolality in patients with nephrogenic diabetes. The mechanism by which thiazide diuretics are able to cause such a paradoxical effect is not well understood. This systematic review seeks to shed light to the details of the exact mechanism through which thiazides and thiazide-like drugs act in patients with DI, so as to bring positive changes in their overall health status and keep the water loss to a minimum.

Completed clinical trials, from January 1965 to January 2025, were included in the systematic review. Risk of bias was evaluated in each of the selected articles. The revised Cochrane risk-of-bias tool for randomized trials (RoB 2) with was used for the two crossover randomized controlled trials (RCTs) and the Risk of Bias In Non-randomized Studies - of Interventions, Version 2 (ROBINS-I V2) assessment tool was used to assess the risk of bias in the non-randomized controlled trials (NRCTs) included in the systematic review. The extensive literature search through PubMed, Cochrane Central Register of Controlled Trials (CENTRAL), and Trip databases resulted in a total of 524 articles screened for this systematic review. Ultimately, only five articles, including two RCTs and three NRCTs were included in this systematic review. It is evident that the counterintuitive use of thiazide diuretics in cases of diabetes insipidus is a tried, tested, and proven method to aid in the management of fluid control in such patients, with notable improvements being identified in urine osmolality as well as urine production over a 24-hour period. The exact mechanism by which each species of thiazide diuretic specifically brings about this paradoxical effect is not well established, but the ultimate pathway by which it reduces the urine output is via proximal tubular reabsorption of water and sodium.

## Introduction and background

Diabetes insipidus (DI) is predominantly characterized by polyuria, polydipsia and dehydration. It is an endocrine disorder defined as follows: An excess of 3L of urine excretion in an adult over a 24-hour period, with the urine osmolality being less than 300 mosm/kg H_2_O. DI is classified into four types, including pituitary, nephrogenic, dipsogenic and gestational forms, with the initial two being the most common [1]. Increased water loss from the body can lead to significant complications, including death, from the severe dehydration, and hence, timely treatment of the disease to prevent and replace the fluid loss should be instituted [1].

In nephrogenic DI (NDI), insults to the distal convoluted tubules (DCT) and collecting ducts, commonly due to the long term use of lithium, leads to a reduced sensitivity to anti-diuretic hormone (ADH). Thiazides and thiazide-like drugs are first-line in its pharmacotherapy [1,2]. In central DI, lesions located in the brain lead to a decreased production of ADH, which is a posterior pituitary hormone. Hence, its first-line treatment is desmopressin (synthetic ADH/ vasopressin/desmopressin acetate (DDAVP)), while thiazide and thiazide-like drugs are a second-line treatment and are used most commonly in mild cases or where patients cannot tolerate the former drug [1,2]. DI can be diagnosed by the indirect water deprivation test or by the copeptin measurement. Initially designated as a diuretic drug, thiazides have primarily established their role in the treatment of essential hypertension [3]. These drugs act by blocking sodium-chloride channels in the DCT, causing a decrease in plasma volume and, eventually, a fall in the total peripheral resistance. Through this mechanism, excess water and salt are excreted through the urine along with some potassium ions, and a lowered blood pressure is consequently attained [3].

When the same drug was trialled in rats with DI in the late 1950s, Crawford and Henry uncovered the potential of thiazides to act paradoxically in this particular situation, in which there is increased water loss through the urine [4]. Thiazides and thiazide-like drugs have shown promising results in reducing the volume of urine output by as much as 50% in DI, especially the nephrogenic type [2,4]. Even in central DI, starting an infant on chlorothiazide and low renal solute load before transitioning to desmopressin has been shown to have better outcomes [5]. Thiazides and thiazide-like drugs have shown promising results in reducing the volume of urine output by approximately 30%, and also increasing urine osmolality in patients with DI, especially the nephrogenic type [4,5]. However, the mechanism through which thiazide and thiazide-like drugs act to decrease the water loss and to increase the urine osmolality in DI, instead of increasing the urine output as it does in hypertension, is still not clear.

It is not fully understood how the same pharmacological drug, given in the same doses, can act in a particular way in a hypertensive patient, so as to produce diuresis, but act so paradoxically in a patient with DI as to produce the exact opposite action [6-10]. Thiazide thus acts as a Swiss knife in different medical conditions, and should therefore be used with caution. Only a few trials have been conducted to study this paradox in more detail. Treatment of DI should be done prudently, because any deviation in the pharmacotherapy and diet can cause either severe dehydration, water toxicity or even severe hypokalemia in the patient which can be life threatening. Hence, it is important to know the precise mode of action of the drugs employed in DI management. This systematic review seeks to shed light to the details of the exact mechanism through which thiazides and thiazide-like drugs act in patients with DI, so as to bring positive changes in their overall health status and keep the water loss to a minimum.

## Review

Methodology

The search for this systematic review was conducted in three electronic databases, namely, PubMed, Cochrane Central Register of Controlled Trials (CENTRAL) and Trip medical database. The results available were limited owing to the lack of clarity in the understanding of the mechanism of action of the drug, which, as a matter of fact, was the focus of this study. To ensure adequate data was on hand for the systematic review, research papers were screened for a greater time period, spanning from January 1965 to January 2025. The literature review carried out was done in accordance with the Preferred Reporting Items for Systematic Reviews and Meta-Analyses (PRISMA) guidelines, which guaranteed transparency and ensured the articles were aligned with accepted standards [11].

Search strategy

Table 1 depicts the databases through which a comprehensive literature review was conducted using various medical subject headings (MeSH)/keywords along with Booleans and the number of available articles.

**Table 1:** MeSH/keywords along with Booleans and the number of available articles CENTRAL: Central Register of Controlled Trials; MeSH: Medical subject headings

Database	MeSH/Keywords terms along with Booleans used for search	Number of articles screened (title and abstract)
PubMed	("Polycystic Kidney Diseases"[Mesh]) AND "Thiazides"[Mesh] Filter: Year 1965-2025	11
PubMed	("Thiazides/pharmacology"[Mesh]) AND "Diabetes Insipidus"[Mesh] Filter: Year 1965-2025	69
PubMed	("Polycystic Kidney Diseases"[Mesh]) AND "Thiazides"[Mesh] ("Thiazides/pharmacology"[Mesh]) AND "Diabetes Insipidus"[Mesh] ((“Diabetes Insipidus”) OR (“Wolfram Syndrome”) OR (“Diabetes Insipidus, Neurogenic”) OR (“Diabetes Insipidus, Nephrogenic”)) AND (“Thiazides” OR “Benzothiadiazines” OR “Bendroflumethiazide” OR “Chlorothiazide” OR “Cyclopenthiazide” OR “Diazoxide” OR “Hydroflumethiazide” OR “Methyclothiazide” OR “Polythiazide”) Filter: Year 1965-2025	294
Cochrane CENTRAL	“Thiazides” AND “Diabetes Insipidus” Filter: Year 1965-2025, Trials	3
Cochrane CENTRAL	“Thiazides” AND “Polycystic Kidney Diseases”	4
Trip medical	((“Diabetes Insipidus”) OR (“Wolfram Syndrome”) OR (“Diabetes Insipidus, Neurogenic”) OR (“Diabetes Insipidus, Nephrogenic”)) AND (“Thiazides” OR “Benzothiadiazines” OR “Bendroflumethiazide” OR “Chlorothiazide” OR “Cyclopenthiazide” OR “Diazoxide” OR “Hydroflumethiazide” OR “Methyclothiazide” OR “Polythiazide”) Filter: Year 1965-2025	143

More than one search query had to be generated due to scarcity of literature, in order to have an adequate number of relevant articles. Each search query consisted of keywords/MeSH terms and boolean operators. The queries were: ("Polycystic Kidney Diseases"[Mesh]) AND "Thiazides"[Mesh]; ("Thiazides/pharmacology"[Mesh]) AND "Diabetes Insipidus"[Mesh]; ((“Diabetes Insipidus”) OR (“Wolfram Syndrome”) OR (“Diabetes Insipidus, Neurogenic”) OR (“Diabetes Insipidus, Nephrogenic”)) AND (“Thiazides” OR “Benzothiadiazines” OR “Bendroflumethiazide” OR “Chlorothiazide” OR “Cyclopenthiazide” OR “Diazoxide” OR “Hydroflumethiazide” OR “Methyclothiazide” OR “Polythiazide”); and “Thiazides” AND “Diabetes Insipidus”.

Inclusion criteria

Completed clinical trials, from January 1965 to January 2025, were included in the systematic review. Full-text articles in the English language were only included. Randomized controlled trials (RCTs) were screened through the RCT filter available in the databases. Non-randomized controlled trials (NRCTs) were hand searched in each database. The articles were selected on the basis of their specific relevance to the anti-diuretic function of thiazide and thiazide-like drugs, including their proposed mechanism of action in different conditions, such as DI and polycystic kidney disease.

Exclusion criteria

Abstracts, case-control studies, cohort studies, cross-sectional studies, case series, case studies, case reports, editorials, viewpoints and letters to the editor/correspondence manuscripts were excluded in this study. Articles in languages other than English were excluded from this review. The exclusion criteria also consisted of incomplete trials, trials whose full text were unavailable, articles whose main focus was on drugs other than thiazide/thiazide-like or which did not emphasize on the mechanism of action of thiazide/thiazide-like drugs and also trials that had led to inconclusive results.

Data extraction

The articles were screened from the three different databases: PubMed, Cochrane and Trip databases. Each article was assessed according to its title, followed by evaluation of its abstract by DR and ALAP, two independent researchers. Consequently, relevant articles were selected according to the inclusion and exclusion criteria as stated above, after going through the full text. The following details of each chosen study were included in the data extraction table: author/year, title, study design, sample size, intervention, daily dose, mechanism of action of drug, outcome and limitation.

Risk-of-bias assessment

Risk of bias was evaluated in each of the selected articles. The revised Cochrane risk-of-bias tool for randomized trials (RoB 2) with additional considerations for crossover trials, was used for the two crossover RCTs included in the systematic review. The Risk of Bias In Non-randomized Studies - of Interventions, Version 2 (ROBINS-I V2) assessment tool was used to assess the risk of bias in the NRCTs selected. The risk of bias was demonstrated through traffic-light plot and weighted bar plots, which were generated using the Risk-of-bias VISualization (robvis) tool.

Results

**Figure 1:**
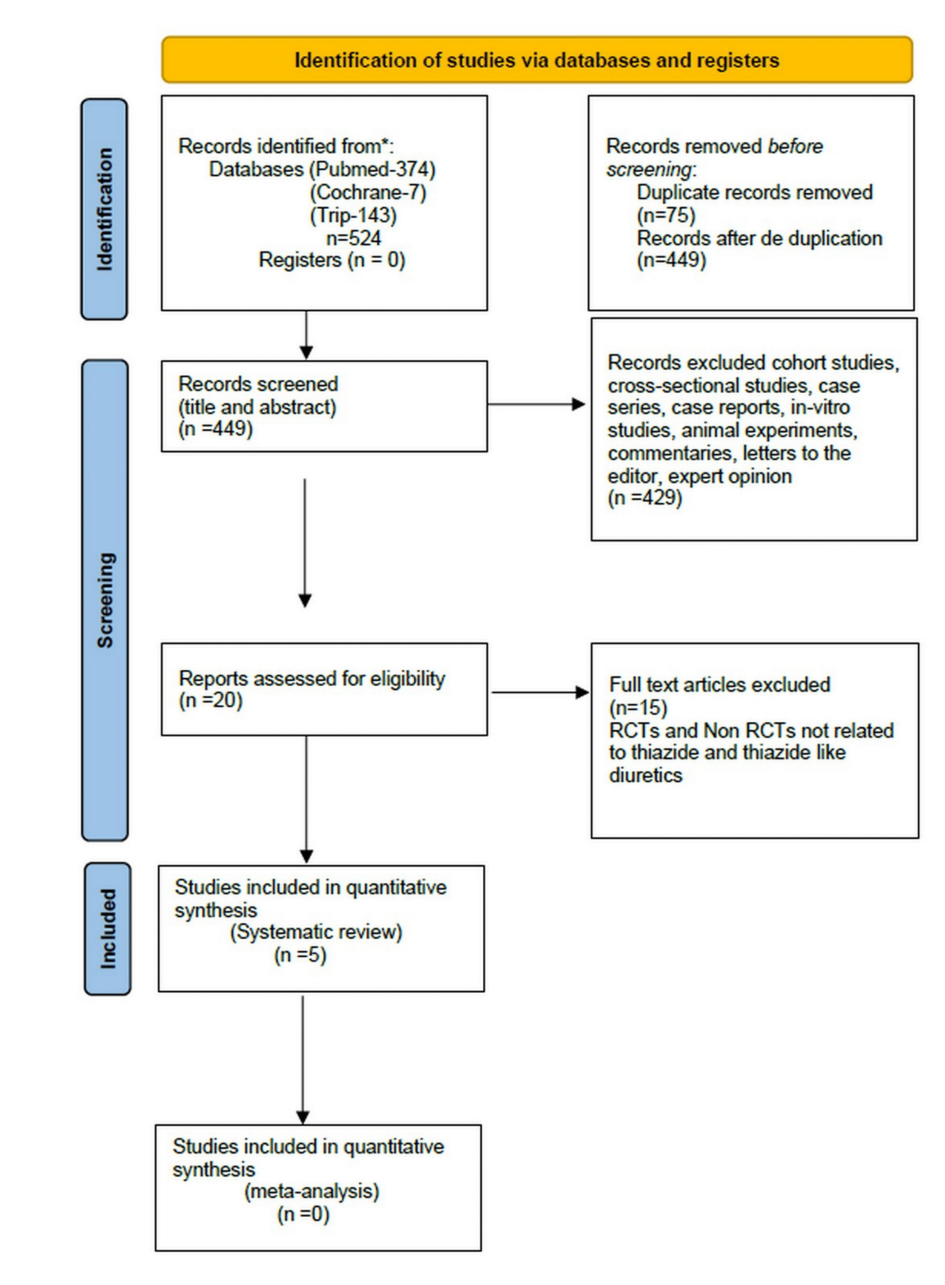
PRISMA 2020 flow chart PRISMA: Preferred Reporting Items for Systematic Reviews and Meta-Analyses

The extensive literature search through PubMed, Cochrane CENTRAL, and Trip databases resulted in a total of 524 articles screened for this systematic review. Of the 524 articles, 75 duplicates were excluded. From the remaining 449 articles, abstracts, case-control studies, cohort studies, cross-sectional studies, case series, case studies, case reports, editorials, viewpoints and letters to the editor/correspondence manuscripts, which added up to 429, were omitted from the study. Consequently, the full text of the 20 articles left were studied in depth and assessed for their eligibility. Finally, only five articles, including two RCTs and three NRCTs were suitable for this systematic review (Figure 1).

The PRISMA flow chart depicts the screening process of the articles up to their selection, from n=524 to n=5.

**Figure 2:**
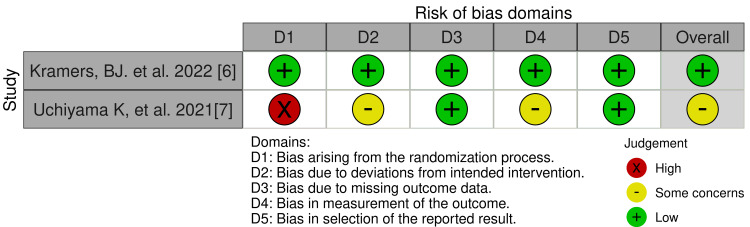
Traffic plot for RCTs RCT: Randomized controlled trial

**Figure 3:**
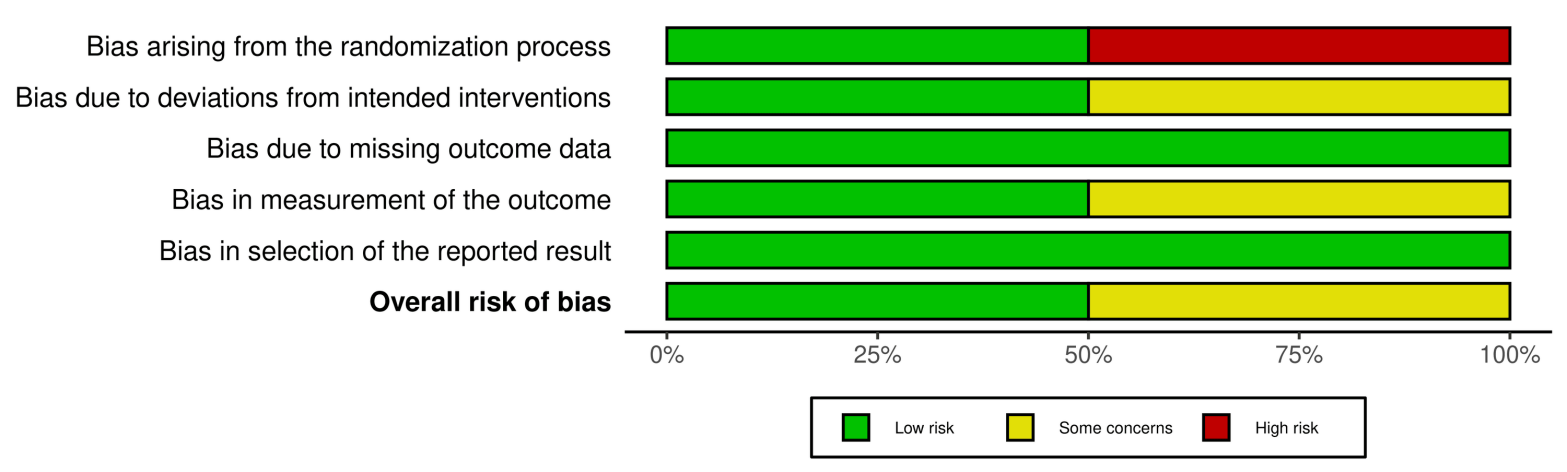
Summary plot for RCTs RCT: Randomized controlled trial

Figures 2, 3 demonstrate the risk of bias in the two RCTs included. RoB 2 with additional considerations for crossover trials was used for the risk assessment. Five domains were considered which were as follows: bias arising from the randomization process (50% high risk), bias due to deviations from intended intervention (50% some concerns), bias due to missing outcome data (100% low risk), bias in outcome measurement (50% moderate concerns), and bias in selection of reported result (100% low risk). The robvis tool was used to generate the traffic plot (Figure 2) and summary/weighted bar plot (Figure 3), which depicts the risk of bias. The overall risk of bias was at 50% low risk and 50% with some concerns.

**Figure 4:**
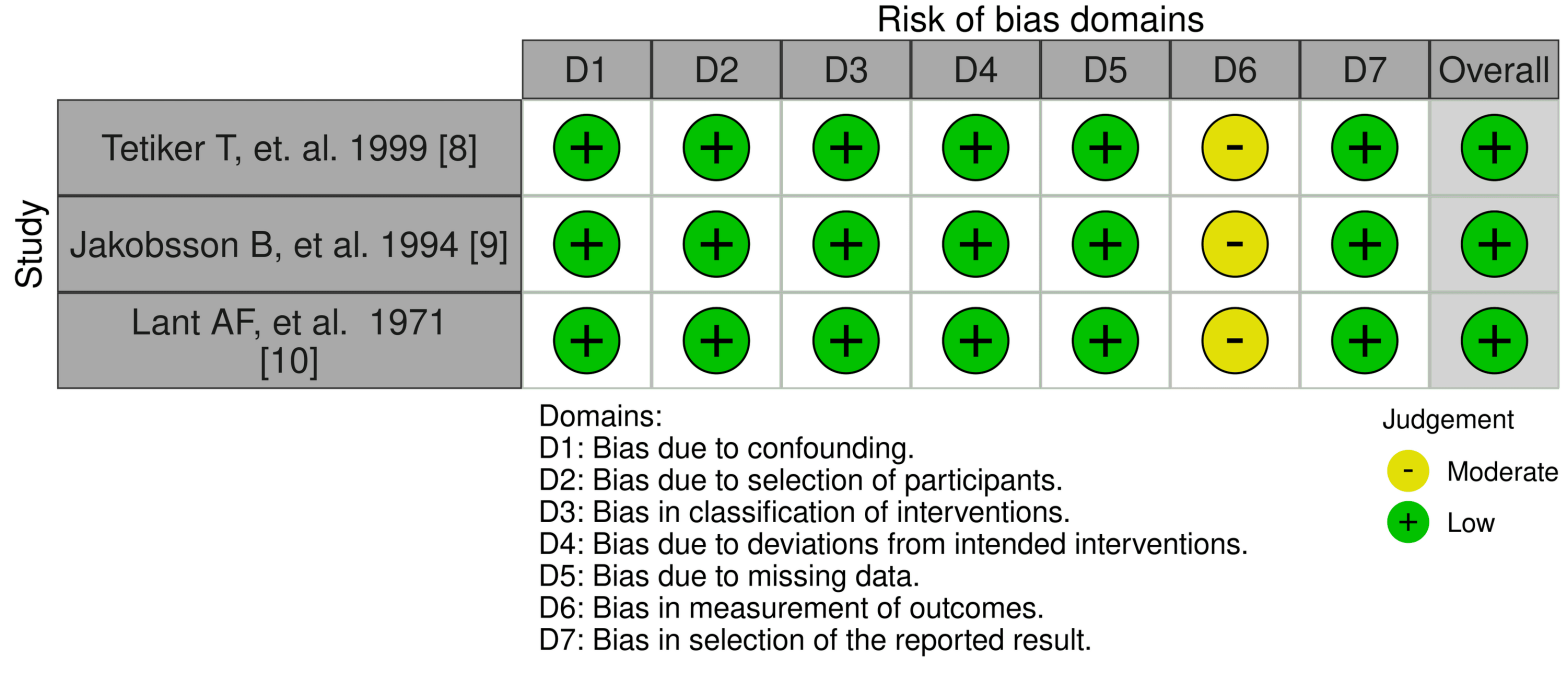
Traffic plot for NRCTs NRCT: Non-randomized controlled trial

**Figure 5:**
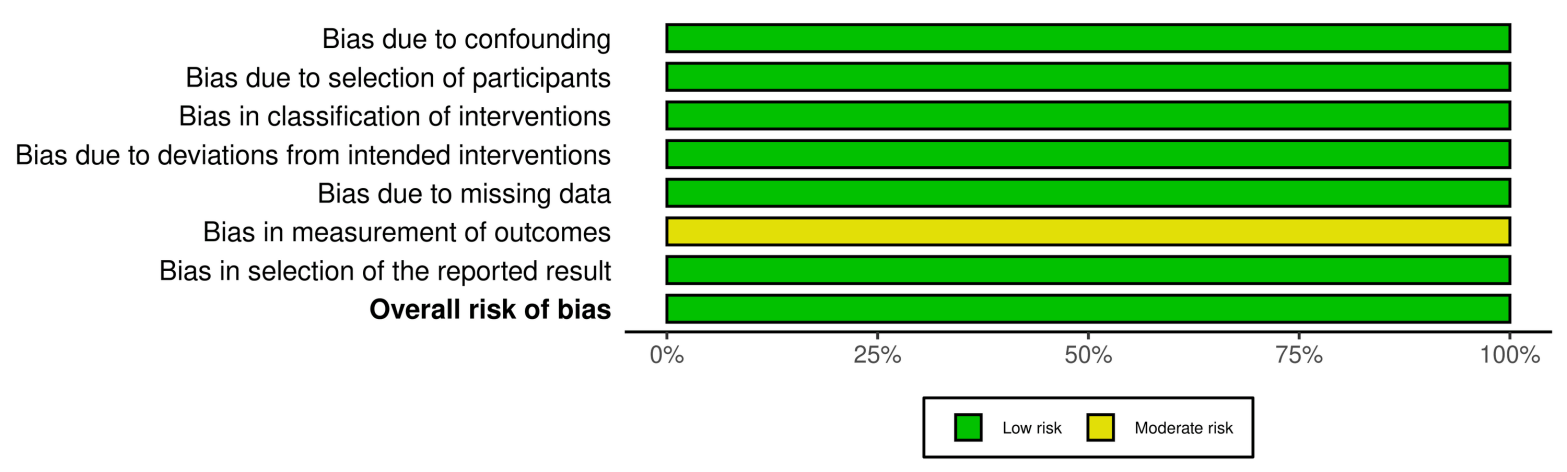
Summary plot for NRCTs NRCT: Non-randomized controlled trial

Figures 4, 5 are traffic-light plot and weighted bar plot, respectively, demonstrating the risk of bias in the three NRCTs. The visualization of the bias was done by the robvis tool after evaluation of the risk of bias using ROBINS-I V2. Seven domains were considered in the assessment: bias due to confounding (100% low risk), bias due to selection of participants (100% low risk), bias in classification of interventions (100% low risk), bias due to deviations from intended interventions (100% low risk), bias due to missing data (100% low risk), bias in measurement of outcomes (100% with moderate concerns), and bias in selection of the reported result (100% low risk). The overall risk of bias was good, at 100% low risk.

Table 2 depicts the author/year, country of study, study design, sample size, the number of intervention patients, the number of control group patients, intervention and daily drug dose. Table 3 depicts the author/year, inclusion criteria, outcome, mechanism of action of the drug and limitations of the study conducted.

**Table 2:** Characteristics of the studies [6-10] NRCT: Non-randomized controlled trial; RCT: Randomized controlled trial

Author/ Year	Country of study	Study design	Sample size	Number of intervention patients	Number of control group patients	Intervention	Daily drug dose
Kramers et al., 2022	The Netherlands	Double-blind crossover RCT	13	13	13	Hydrochlorothiazide	Week 1: 12.5mg and Week 2: 25mg
Uchiyama et al., 2021	Japan	Open-label crossover RCT	10	10	10	Trichlormethiazide	Not available
Tetiker et al., 1999	Turkey	NRCT	20	20	-	Indapamide and chlorpropamide	Indapamide: 2.5mg/day, chlorpropamide: 250 mg/day
Jakobsson et al., 1994	Sweden	NRCT	4	4	-	Hydrochlorothiazide and indomethacin	Hydrochlorothiazide: 2 mg/kg/day, indomethacin: 2 mg/kg/day
Lant et al., 1971	United Kingdom	NRCT	7	7	-	Oral benzothiadiazine	Hydroflumethiazide: 50-100 mg/day, polythiazide: 3-4 mg/day

**Table 3:** Clinical outcome and mechanism of action of drugs [6-10] ADPKD: Autosomal dominant polycystic kidney disease; DI: Diabetes insipidus; eGFR: Estimated glomerular filtration rate; GFR: Glomerular filtration rate; HRQOL: Health-related quality of life; KDQOL: Kidney disease quality of life; NDI: Nephrogenic diabetes insipidus; NRCT: Non-randomized controlled trial; RCT: Randomized controlled trial

Author/year	Inclusion criteria	Outcome	Mechanism of action of drug	Limitations
Kramers et al., 2022	Patients 18-50 years of age, diagnosed with ADPKD with an eGFR ≥45 ml/min per 1.73 m^2^ and being treated with tolvaptan.	A significant decrease was observed in 24-hr urine volume with hydrochlorothiazide, at 5.1 L/24 hours (p < 0.0001), and in plasma copeptin level at 5.6 pmol/L (95% confidence interval, −9.0 to −2.3 as compared to placebo (p<0.0001). Mild decrease in GFR noted (55±11 to 51±10 ml/min per 1.73 m^2^).Increase in urine osmolality was noted.	The drug induces a decrease in extracellular volume, leading to increased sodium reabsorption in proximal tubule and lesser volume of preurine reaching the collecting duct.	Small sample size. Baseline measurements were not recorded again after washout periods before the next drug (intervention/placebo) was administered. Few cases of mild hypokalemia seen.
Uchiyama et al., 2021	Patients 44-71 years old, with ADPKD and average eGFR = 39.3ml/min per 1.73 m^2^, with drug-induced block of vasopressin V2 receptor and receiving a high dose of tolvaptan (>60mg/day).	Decreased urinary volume (3,348±584 mL from 4,255±739 mL/day) (p< 0.001). Serum osmolality and increased urine osmolality was noted. Decrease in plasma copeptin and plasma vasopressin. Increase in eGFR slope. Improvement of few HRQOL subscales.	Increased sodium and water reabsorption in the proximal tubule, induced by hypovolemia. This is followed by lower volume of water and sodium reaching the collecting ducts and lesser urine formed.	Small sample size. More risk of bias since study was not blinded. Some carry-over effects might still be present.
Tetiker et al., 1999	Patients aged 16-55 years, suffering from central DI. The urine volume ranged from 4,700mL/day to 12,700mL/day, and urine osmolality 83-303 mosm/kg H_2_O.	Indapamide: Decrease in urinary output by an average 40%. The mean±SD reduction was 40.56%±9.70% (range: 19.6%-55.0%) (p<0.05). Increase in urinary osmolality in 9 patients and decrease serum osmolality in 4 others. No side effects. Chlorpropamide: 48% decrease in urine output. Urinary osmolality increased in 7 patients and serum osmolality decreased in 7 patients.	Indapamide has a chloro sulfamoyl benzamide moiety as present in thiazides. It causes proximal tubular reabsorption of water and sodium and hence decreased urine output.	Small sample size. Trial is non-randomized and not blinded. Chlorpropamide caused severe hypoglycemia in 2 patients.
Jakobsson et al., 1994	Patients aged 5-12 years, diagnosed with NDI. One pair of brothers had true NDI and the other set had partial NDI.	Decrease in mean 24-hr urine output by 25% with hydrochlorothaizide and additional 17% decrease when given with indomethacin (p< 0.01). Slow increase in urine osmolality. Increase in plasma renin level in true NDI patients with hydrochlorothiazide, normalized after adding the second drug. GFR remained normal. Increased plasma aldosterone level with both hydrochlorothiazide and indomethacin. Increased medullary osmotic content.	Volume and sodium depletion in NDI is followed by proximal tubular reabsorption of sodium and, consequently, there is decreased urine flow.	Small sample size and trial carried in non-randomized population. The participants each had a different spectrum of the same disease.
Lant et al., 1971	Patients aged 9-52 years, suffering from pituitary DI. Initial fluid turnover ranging from 4.95L/24 hrs to 17.20L/24 hrs and initial serum osmolality 292-301 mosm/kg H_2_O.	Immediate: Decrease in urinary volume 30±10 % and increase in urine osmolality. Slight decrease in GFR. Less thirsty. Long term: Maintenance of antidiuresis action and decreased thirst. Decrease in osmolality was restored. Rise in serum uric acid.	Administration of the drug initially causes a rise in circulating renin and angiotensin, subsequently leading to an increased sodium reabsorption in the proximal tubule and, hence, a decreased urine output.	Small sample size. Trial is not blinded and non-randomized. Long-term: Asymptomatic hypokalemia, mild alkalosis.

Discussion

DI presents with the main symptom of polyuria with urine that can be normal or diluted. Though being of two main types, namely, central DI (inadequate production of ADH) and NDI (unresponsiveness to ADH), the primary aim of the treatment remains the maintenance of hydration by preventing a fluid imbalance [12]. While the first line of treatment is usually administration of the reduced hormone ADH, thiazide, a diuretic has long been in the repertoire of drugs for DI despite the fact that there is limited information available about its use in this case. It exerts a counterintuitive action as an anti-diuretic and improves the polyuric state.

A clinical trial conducted by Lant et al. studied the use of hydrochlorothiazide in the treatment of DI over a long period [10]. Based on the inclusion criteria, the effect of administering a benzothiadiazine or phthalimidine diuretic to seven patients suffering from central DI were investigated. The diuretics administered to those patients included hydroflumethiazide (50-100 mg/day) and polythiazide (3-4 mg/day); clorexolone (25-50 mg/ day); and potassium sparing diuretics triamterene (100 mg/day) and amiloride (10 mg/day), respectively. Patients were provided with the necessary equipment and were discharged to self-monitor urine at home. They had to report to a follow-up clinic once or twice in two months.

The study revealed a prompt decrease of 30±10% in the volume of urine as from Day 1 with a simultaneous increase in the urine concentration with all the drugs [10]. In the instance where minimum doses of the drug were used, the anti-diuretic effect increased proportionally as the doses were incremented. The anti-diuretic action was not only immediate but, polythiazide had a residual action of up to one week post therapy [10]. This immediate anti-diuretic action is thought to be secondary to a rise in salt diuresis affecting mainly the concentration of the urine [6,7,9,10]. However, as supported by Earley et al., long-term administration of thiazide and thiazide-like drugs for DI resulted in not only better urine concentrating capacity but also a significant decrease in urinary volume [13].

The diuretic-induced antidiuresis is not limited to benzthiazides; thiazide-like drugs have also been used in the management of DI [10]. Tetiker et al. studied the therapeutic potential of indapamide, a thiazide-like diuretic, in DI [8]. 20 consecutive patients suffering from central DI received indapamide in the dose of 2.5 mg daily over 10 days after baseline level of serum and urine osmolality, urine volume, and serum electrolytes were charted. For the sake of comparison, after 10 days of therapy, 11 out of the 20 patients were administered chlorpropamide daily and the same variables were monitored. Evaluation of the 24-hour urinary volume after treatment with indapamide demonstrated a mean±SD reduction of 40.56±9.70%. It is worth noting that none of the patient experienced side effects important enough to discontinue the study compared with chlorpropamide where 2 patients suffered from severe hypoglycemia [8]. As Gonzalez et al. had reported the use of indapamide in the treatment of a case of DI in absence of vasopressin, it demonstrates the therapeutic capacity of the drug’s anti-diuretic action [14].

Jakobsson et al. performed a study to evaluate the efficacy of hydrochlorothiazide either alone or in combination with indomethacin in four patients (two pairs of brothers) with NDI [9]. Two of the patients suffered from partial NDI with capacity to concentrate urine on taking desmopressin, while the other two had true NDI. The study started after a wash-out period of at least two weeks after which the drugs were administered. Investigations were performed on three occasions over a period of one month within which hydrochlorothiazide (2 mg/kg/day) was administered as a monotherapy for the first two weeks, and an adjunct of indomethacin was added in the second two weeks of the study. On the three occasions, a 24-hour urine sample was collected to measure the volume, serum, and urinary osmolality and electrolytes. Moreover, the change in urinary volume was associated with an improvement in the renal capacity to concentrate urine on both occasions. Findings from a study by Vierhapper et al. similarly validated the fact that hydrochlorothiazide definitely has anti-diuretic action in DI and hydrochlorothiazide-indomethacin co-administration produced a greater reduction in urinary volume [15].

An open-label, randomized, controlled, counterbalanced, crossover trial conducted by Uchiyama et al. treated 10 known cases of autosomal dominant polycystic kidney disease (ADPKD) suffering from tolvaptan-induced polyuria by administration of trichlormethiazide [7] . Based on the inclusion criteria, 14 patients were selected, out of which 10 consented to be study participants. The study samples were randomized with five patients receiving trichlormethiazide and the remaining five receiving no drugs. This was followed by a crossover study and the intervention was completed over a total period of 24 weeks. The mean urinary volume when taking trichlormethiazide showed a marked decrease compared to in its absence (3,348 ± 584 vs. 4,255 ± 739 mL) [7]. The mean urinary osmolality also was positively affected by revealing an increase in the urine concentrating ability of the kidneys. The positive effect of trichlormethiazide in combination with tovalptan in ADPKD is expected to not only increase compliance to the treatment but to also improve the quality of life of such patients [7]. However, as mentioned by Kramers et al., further studies need to be conducted to meticulously assess the drug-drug interaction of trichlormethiazide and Tovalptan in the case of co-prescription [16].

Kramers et al. similarly investigated the effect of hydrochlorothiazide on diuresis induced by the established pharmacotherapy of known cases of ADPKD [6]. The randomized, controlled, double-blind, crossover trial studied 13 patients of ADPKD currently being treated with tolvaptan. They were randomized and administered hydrochlorothiazide (25 mg OD), metformin (1,000 mg BD), and placebo in a random order over a period of eight weeks, with a one-week gap between each drug. 24-hour urine samples were collected every two weeks after drug administration. It revealed a reduction in pre-therapy urinary volume from 6.9±1.4 L/24 hours to 5.1±1.5 L/24 hours with hydrochlorothiazide. Post-metformin therapy also revealed a decrease in the volume of urine while minimal change was noted with the placebo. However, hydrochlorothiazide had a higher degree of tolerability compared to that of metformin. The study depicted that there was no significant raise in the molecules, implying renal damage occurred with the acute administration of metformin, but it failed to however prove its nephroprotective aspect [6]. A study by Tas et al. shone light on metformin’s capacity to reduce oxidative stress reactants, which could be seen through histopathological studies of the kidneys in rats [17]. However, the nephroprotective effect of metformin in humans remains uninvestigated specifically in DI, even though positive findings have been shown in diabetes mellitus. The study, thus, favors the use of thiazide diuretics in the pharmacotherapy of NDI [6,18].

The current limitation of this study is that there are only few studies addressing thiazide as an anti-aquaretic in the pharmacological therapy of DI. Moreover, studies carried out to bridge the gap of knowledge, have the constraints of small study sample sizes. Further studies need to be carried out on a larger scale yielding recent breakthroughs to meet the dearth of information in paradoxical action of thiazide in DI, which would reshape and improve the medical care in DI.

## Conclusions

It is evident that the counterintuitive use of thiazide diuretics in cases of DI is a well-established approach in the management of fluid control. This strategy is found to be effective in reducing urine osmolarity and urinary output in such patients. The exact mechanism by which each species of thiazide diuretic specifically brings about this paradoxical effect is not well established. The ultimate pathway by which it reduces the urine output is via a rise in circulating renin and angiotensin. This, in turn, induces proximal tubular reabsorption of water and sodium as a compensatory response to hypovolemia, thereby reducing urine output. 
